# Factors predicting composite grafts survivability in patients with fingertip amputations; a systematic review and meta-analysis

**DOI:** 10.1186/s13018-024-05230-9

**Published:** 2024-11-18

**Authors:** Ali Mohamed Elameen, Asmaa Ali Dahy, Ahmed Abu-Elsoud, Amany Attalah Gad

**Affiliations:** 1 Department of Plastic and Reconstructive Surgery, El-Sahel Teaching Hospital, Cairo, Egypt; 2 Department of Plastic and Reconstructive Surgery, Faculty of Medicine For Girls, Al-Azhar University, Nasr City, Cairo, Egypt; 3 Department of Plastic and Reconstructive Surgery, Faculty of Medicine (Assiut Branch), Al-Azhar University, Cairo, Egypt

**Keywords:** Composite graft, Survival, Fingertip, Amputations

## Abstract

**Background:**

Fingertip amputation is a commonly encountered injury in emergency settings. Composite grafting is a non-microsurgical alternative maintaining digit length with no donor site morbidities. This meta-analysis was conducted to retrieve factors associated with composite graft survivability among patients with fingertip amputations.

**Methods:**

A literature review throughout twelve databases was performed on 24 July 2023. All clinical studies comparing the patients-related, trauma-related, or amputation-related variables among patients with survived and non-survived composite grafting were eligible for meta-analysis. Single-arm studies reported the potential predictors of composite graft survival among patients with fingertip injuries treated with composite grafting were included.

**Results:**

This review included ten articles with 720 fingertips composite grafting. Of them, 526 grafts survived, with a pooled overall survivability of 72.8%. There was a significant association between younger age (OR 2.31,95%CI 1.10, 4.87, *P* = 0.03), level of amputation (I) (OR 0.31,95% CI 0.14 to 0.67, *P* = 0.003), and successful composite grafting. There was no statistically significant (*P* = 0.449) impact of time to composite grafting on the likelihood of composite graft survivability.

**Conclusion:**

Composite grafting is a feasible and effective procedure for restoring aesthetically functional digits among patients with traumatically amputated fingertips. The composite graft survived among the majority of the patients, with a more significant survival pattern among younger populations and patients with more distal amputations.

**Level of evidence:**

Level III.

**Supplementary Information:**

The online version contains supplementary material available at 10.1186/s13018-024-05230-9.

## Introduction

Fingertip amputation is a commonly encountered injury in emergency settings. It is a segmental loss of the digits distal to the distal interphalangeal joint. It accounts for approximately two-thirds of all hand injuries among the pediatric population [1]. Crush injuries are the most attributable cause of digital tip amputations among pediatrics. Fingertip amputations are associated with debilitating functional and psychological consequences. This includes poor sensory recovery, hypersensitivity, cold intolerance, and infection. Furthermore, patients may experience multiple finger deformities and disfigurements, resulting in long-term disabilities [2, 3]. The ideal interventions for fingertip amputations should restore minimally shortened and painless fingers with sensate and durable skin coverage with preserved functional and cosmetic outcomes [4].

Fingertip replantation is the ideal procedure for fingertip amputations. It re-establishes immediate vascularity to the compromised amputated fingertip, maintaining a stable and durable digit [5]. However, this demanding technique necessitates particular equipment, prolonged surgical experience, and well-prepared infrastructures. Furthermore, the complex vascularity of the distal phalanx makes replantation techniques impossible in certain circumstances [6]. This includes too distal amputations, crushing injury, avulsion injury, or inadequate vessel state for vascular anastomosis. Non-microsurgical techniques provide a feasible alternative to fingertip replantation in non-replantable settings. This includes healing by secondary intention, skin grafts, loco-regional flaps, or repositioning of the amputated part as a composite graft [7]. Composite grafting is a non-microsurgical alternative to maintain the digit length without donor site morbidities. The successful rate of complete composite grafting survivability is nearly 17%, while partial graft taken is approximately 81% among the pediatric population [8]. It is a simple, cost-effective, and time-effective procedure that preserves the digital length and partially restores the motor and sensory functions of the finger with a near-normal nail complex architecture. However, the composite graft is associated with considerable adverse events such as infection, necrosis, color mismatches, and poor sensibility [9].

The composite graft is an alternative option to reconstruct a non-replantable amputated fingertip. However, there is significant controversy to which factors enhance composite graft success after fingertip amputations. Previous studies highlighted the impact of the level of injury, time to grafting, amputation mechanism, smoking status, and comorbidities. However, the findings of previously published studies are inconsistent with the difference in survivability rate [10, 11]. Therefore, this systematic review and meta-analysis was conducted to retrieve the patients-related, trauma-related, and amputation-related factors of composite graft survivability among patients with fingertip amputations. Recognizing such evidence is crucial to ensuring a better prognosis and optimizing an individualized decision-based approach for patients with traumatic fingertip amputations.

## Methods

This meta-analysis was performed parallel with the Preferred Reporting Items for Systematic Reviews and Meta-Analysis (PRISMA) guidelines [12] and the recommendations of the Cochrane collaboration [13]. The methodology of the study was registered in the PROSPERO (CRD42023459514) database. (Supplementary Table 1).

### Literature search

The literature was reviewed through twelve databases on 24 July 2023. The following databases were searched using individualized search strings customized for each database: PubMed, ISI, Google Scholar, Scopus, NYAM, SIGLE, VHL, Clinical trials, mRCT, Cochrane Collaboration, EMBASE, and ICTRP. There were no limitations regarding age, gender, the language of publication, ethnicity, definitions of amputations, graft survivability, and study regions. Citation tracking, cross-referencing, and reviewing the references of the eligible articles were carried out to retrieve all possible relevant articles. The following keywords were used; ‘Digits’, ‘Fingertip’, ‘Fingertips’, ‘ ‘Digital Tips’, ‘Fingers’, ‘Digital Tip’, ‘Digit’, ‘Finger’, ‘Thumb’, ‘Thumbs’, ‘Graft’, ‘Grafts’. (Supplementary Table 2).

### Study selection

All clinical studies comparing the patients-related, trauma-related, or amputation-related variables among patients with survived and non-survived composite grafting were eligible for meta-analysis. Single-arm studies reported the potential predictors of composite graft survival among patients with fingertip injuries treated with composite grafting were included. Non-comparative studies or irrelevant articles were ineligible. Studies with unextractable data, review articles, guidelines, comments, case reports, animal studies, letters, posters, books, or editorials were not included.

The articles retrieved from the screening process were exported to an Excel sheet after the initial removal of the duplicated reports using EndNote X9[14]. The screening processes were performed to reveal the finally eligible studies for data extraction.

### Data extraction and quality assessment

The study-related data were retrieved from the articles that were finally analyzed. This included the study ID, study region, study URL, study design, and study period. The methodology-related data were extracted, including the eligibility criteria, diagnostic criteria of amputation levels, population, and follow-up protocol. The number of composite grafts was stratified by the pattern of survivability into complete, partial, and failed survivability. The patients’ related data, encompassing age, gender, and comorbidities, were retrieved. Trauma-related factors were extracted, including mechanism of injury, mode of trauma, time to composite grafting, contamination, affected hand, affected fingers, number of affected fingers, and associated injuries. The amputation-related data were retrieved, including the level of amputation, composition of the amputated part, transport mode, bone exposure, fracture type, and adequate preservation of the amputated part. Management-related data, such as the application of Lipo-prostaglandin, ice-cooling, and hyperbaric oxygen, were retrieved. The time since injury in the survived and failed composite grafts groups was extracted from Murphy et al., 2017 using WebPlotDigitizer software [15]. The quality of the analyzed articles was evaluated using the National Institute of Health (NIH) quality assessment tool [16]. A detailed description of classifications of fingertip amputations is described in the (supplementary Table 3).

### Data analysis

The prevalence of composite graft survival was calculated by pooling the event rate and 95% confidence intervals (CIs) for each study, then calculating the effect sizes of all articles to estimate the overall event rate with 95%CI. Mean difference (MD) was used for calculating the summary effect from continuous variables. Mean and standard deviation (SD) was calculated from mean and range or median and range following equations provided by Hozo et al. [17] The risk ratio (RR) or odds ratio (OR) with 95% CI was used for reporting the dichotomous variables. The fixed-effect model was applied when a fixed population effect size was assumed. On the contrary, the random-effects model was implemented. Statistical heterogeneity was evaluated using Higgins *I*^*2*^ statistics and the Cochrane Q (*Chi*^2^ test) [18]. Meta-analysis was performed using Review Manager version 5.4 (Revman 5.4) and Comprehensive Meta-Analysis v3 (CMA V3) software[19, 20]. The significant difference was revealed when the probability value (*P*) < 0.05.

## Results

**Fig. 1:**
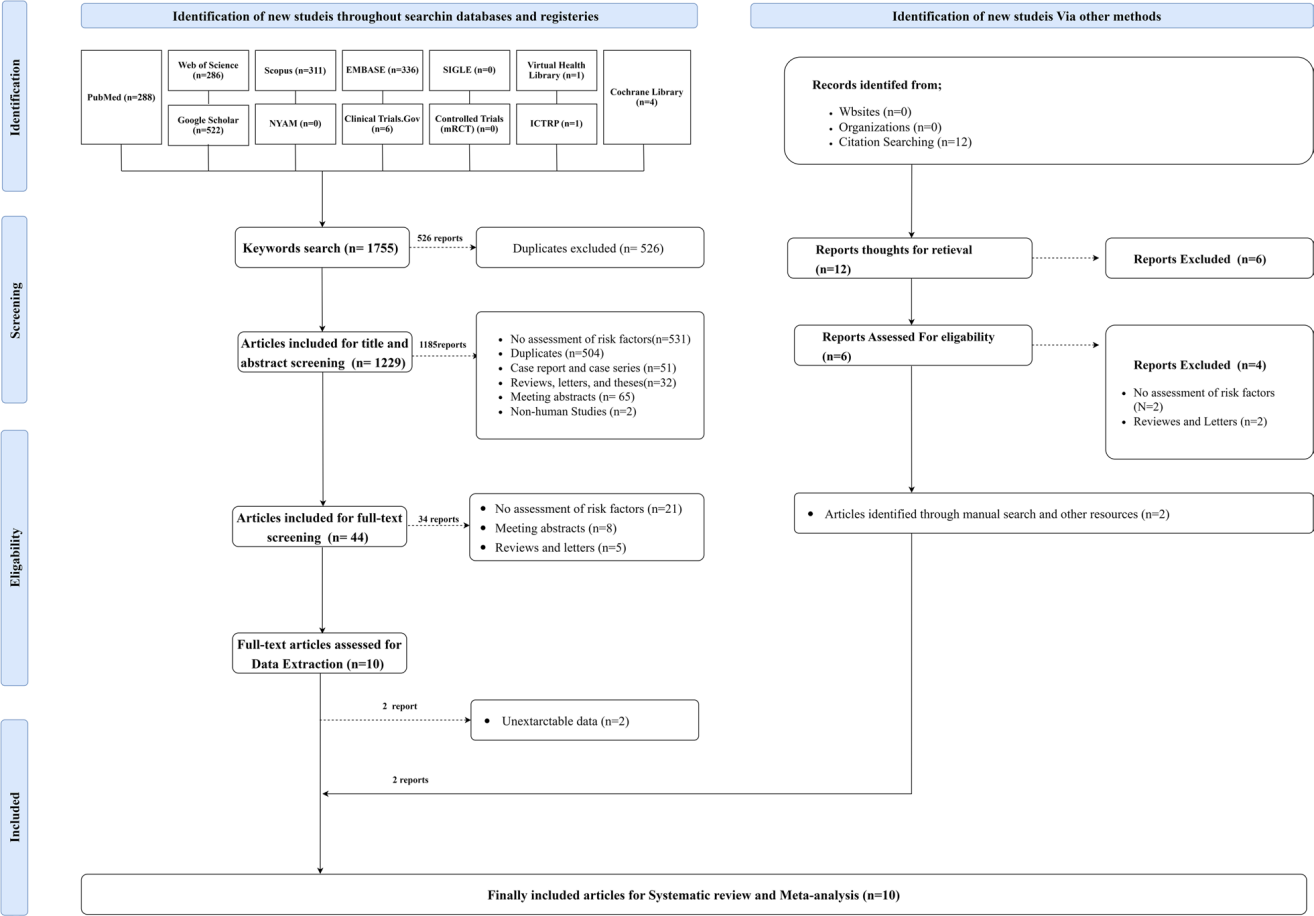
PRISMA 2020 flow diagram for updated systematic reviews which included searches of databases, registers, other sources, and screening

A literature review of twelve databases revealed 1755 potentially relevant articles. Duplicates were initially excluded, retrieving 1229 reports eligible for screening. Out of them, 1185 articles were excluded, resulting in 44 studies included for full-text screening. Thirty-four studies were ousted, yielding ten articles for data extraction. Two reports with unextractable data were excluded, and two were included in the citation tracking method. Ten articles were eventually eligible for systematic reviewing and meta-analysis. (Fig. 1).

### Demographic characteristics of the analyzed articles

The present systematic review included ten articles with 720 fingertips composite grafting [7, 21–29]. There were eight articles of retrospective design and two of prospective design. Four articles included patients from South Korea and three from the United Kingdom. Four studies implemented the Modified-Ishikawa classification for fingertip amputations. Five studies included only the pediatric population; three reports included the adult population. Of the included 720 composite grafts, 526 grafts showed complete or partial composite graft survivability. The average age of the included patients hovered between 4.3 and 46.07 years. There were 274 males and 189 females. The average follow-up period ranged from 8 weeks to 14.8 months. (Table 1).

**Table 1:** Demographic characteristics of the included studies

Study ID		Study region	Study design	Study period	Diagnostic criteria	Study population	Follow-up period	Sample Size		Outcomes		
								Survived	Failed	Complete	Partial	Failed
								Number	Number	Number	Number	Number
1	Borrelli et al., 2018[21]	UK	Retrospective	January 01 2006 and 31 December 2016	Modified-Ishikawa	Pediatric	4.65 ± 10.85 months	59	41	13	46	41
2	Butler et al., 2016[7]	UK	Retrospective	October 2006 and April 2013	Modified-Ishikawa	Pediatric	1.8 months	43	54	10	33	54
3	Eberlin et al., 2015[22]	USA	Retrospective	January 2007 to December 2012	NR	Pediatric	137 days (4.5 months)	26	13	3	23	13
4	Eo et al., 2018 [23]	South Korea	Retrospective	March 2008 and September 2017	Das	Pediatric and Adult Population	3 months	84	10	NR	NR	NR
5	Heistein and Cook, 2003 [24]	USA	Prospective cohort	December 1997 to June 2000	Allen	Pediatric and Adult Population	1 (5–8 days) and 12 weeks after the injury	48	9	30	18	9
6	Lee et al., 2023[25]	South Korea	Retrospective	January 2013 and December 2017	Allen	Adult	8 weeks	28	27	NR	NR	NR
7	Lee et al., 2020 [26]	South Korea	Retrospective	January 2015 and July 2020	Ishikawa	Adult	6 months	121	20	94	27	20
8	Lim et al., 2019 [27]	South Korea	Retrospective	January 2007 and December 2017	Das	Adult	NR	25	16	NR	NR	NR
9	Moiemen and Elliot, 1997 [28]	UK	Prospective cohort	June 1989 to January 1993	Modified-Ishikawa	Pediatric	14.8 months	36	13	11	26	13
10	Murphy et al., 2017[29]	Australia	Retrospective	January 2003 to December 2014	Modified-Ishikawa	Pediatric	NR	56	11	30	26	11

Study ID		Age (Years)		Gender				Smoking		Time Delay > 6 h	
				Males		Females					
		Survived	Failed	Survived	Failed	Survived	Failed	Survived	Failed	Survived	Failed
		Mean ± SD	Mean ± SD	Number	Number	Number	Number	Number	Number	Number	Number
1	Borrelli et al., 2018[21]	NR	NR	32	25	27	16	NR	NR	45	49
2	Butler et al., 2016[7]	4.3 (1–15)*		NR	NR	NR	NR	NR	NR	NR	NR
3	Eberlin et al., 2015[22]	NR	NR	24		15		NR	NR		NR
4	Eo et al., 2018 [23]	40 (1–68)*	37 (25–51)*	60	9	24	1	28	6	15	5
5	Heistein and Cook, 2003 [24]	NR	NR	19		9		2	10	NR	NR
6	Lee et al., 2023[25]	40.93 ± 11.84	46.07 ± 18.00	NR	NR	NR	NR	10	6	NR	NR
7	Lee et al., 2020 [26]	38.4 ± 17.0		74		87		NR	NR	NR	NR
8	Lim et al., 2019 [27]	40.08 (16–65)*	42.19 (20–76)*	16	15	9	1	8	NR	NR	NR
9	Moiemen and Elliot, 1997 [28]	NR	NR	NR	NR	NR	NR	NR	NR	22	10
10	Murphy et al., 2017[29]	NR	NR	NR	NR	NR	NR	NR	NR	37	16

SD, standard deviation; NR, non-reported; UK, United Kingdom; USA, United States of America

*data reported using median and range

There were 336 patients with clean-cut fingertip amputations. The most affected finger was the middle finger, succeeded by the index finger, accounting for 66 and 39 fingertips, respectively. There were 356 patients with fingertip amputations class I and 207 patients with class II fingertip amputations. There were 72 patients with fingertip amputations class III. The quality score of the analyzed retrospective and prospective studies ranged from 60 to 80%, with all articles being good quality apart from Eberlin et al., [22], which showed a fair quality (Table 2).

**Table 2:** Trauma related data and quality assessment of the included studies

Study ID		Mechanism of Injury						Affected Fingers									
		Cut		Avulsion		Crushed		Thumb		Index finger		Middle finger		Ring finger		Little finger	
		Survived	Failed	Survived	Failed	Survived	Failed	Survived	Failed	Survived	Failed	Survived	Failed	Survived	Failed	Survived	Failed
		Number	Number	Number	Number	Number	Number	Number	Number	Number	Number	Number	Number	Number	Number	Number	Number
1	Borrelli et al., 2018[21]	49	26	3	10	7	4	NR	NR	NR	NR	NR	NR	NR	NR	NR	NR
2	Butler et al., 2016[7]	NR	NR	NR	NR	NR	NR	NR	NR	NR	NR	NR	NR	NR	NR	NR	NR
3	Eberlin et al., 2015[22]	2		1		24		1		6		15		9		8	
4	Eo et al., 2018 [23]	58	2			26	8	10	0	20	1	31	5	16	3	7	1
5	Heistein and Cook, 2003 [24]	10	6	11	7	7	12	NR	NR	NR	NR	NR	NR	NR	NR	NR	NR
6	Lee et al., 2023[25]	25	9			2	16	NR	NR	NR	NR	NR	NR	NR	NR	NR	NR
7	Lee et al., 2020 [26]	128		33				NR	NR	NR	NR	NR	NR	NR	NR	NR	NR
8	Lim et al., 2019 [27]	18	3	NR	NR	7	13	2	2	8	4	9	6	4	1	2	3
9	Moiemen and Elliot, 1997 [28]	NR	NR	NR	NR	NR	NR	NR	NR	NR	NR	NR	NR	NR	NR	NR	NR
10	Murphy et al., 2017[29]	NR	NR	NR	NR	NR	NR	NR	NR	NR	NR	NR	NR	NR	NR	NR	NR

Study ID		Level of amputation								Quality assessment	
		1a		1b		II		III			
		Survived	Failed	Survived	Failed	Survived	Failed	Survived	Failed		
		Number	Number	Number	Number	Number	Number	Number	Number	%	Decision
1	Borrelli et al., 2018[21]	3	0	14	12	26	16	8	8	80	Good
2	Butler et al., 2016[7]	4	8	26	25	12	20	1	1	70	Good
3	Eberlin et al., 2015[22]	NR	NR	NR	NR	NR	NR	NR	NR	60	Fair
4	Eo et al., 2018 [23]	43		1		26	5	15	4	80	Good
5	Heistein and Cook, 2003 [24]	NR	NR	NR	NR	NR	NR	NR	NR	80	Good
6	Lee et al., 2023[25]	12		9		13	13	3	5	70	Good
7	Lee et al., 2020 [26]	77	9	35	9	17	6	2	9	80	Good
8	Lim et al., 2019 [27]	14		3		6	4	5	9	80	Good
9	Moiemen and Elliot, 1997 [28]	NR	NR	NR	NR	NR	NR	NR	NR	70	Good
10	Murphy et al., 2017[29]	14	2	32	4	10	3	0	2	70	Good

NR, non-reported

### Prevalence of composite graft survival

**Fig. 2:**
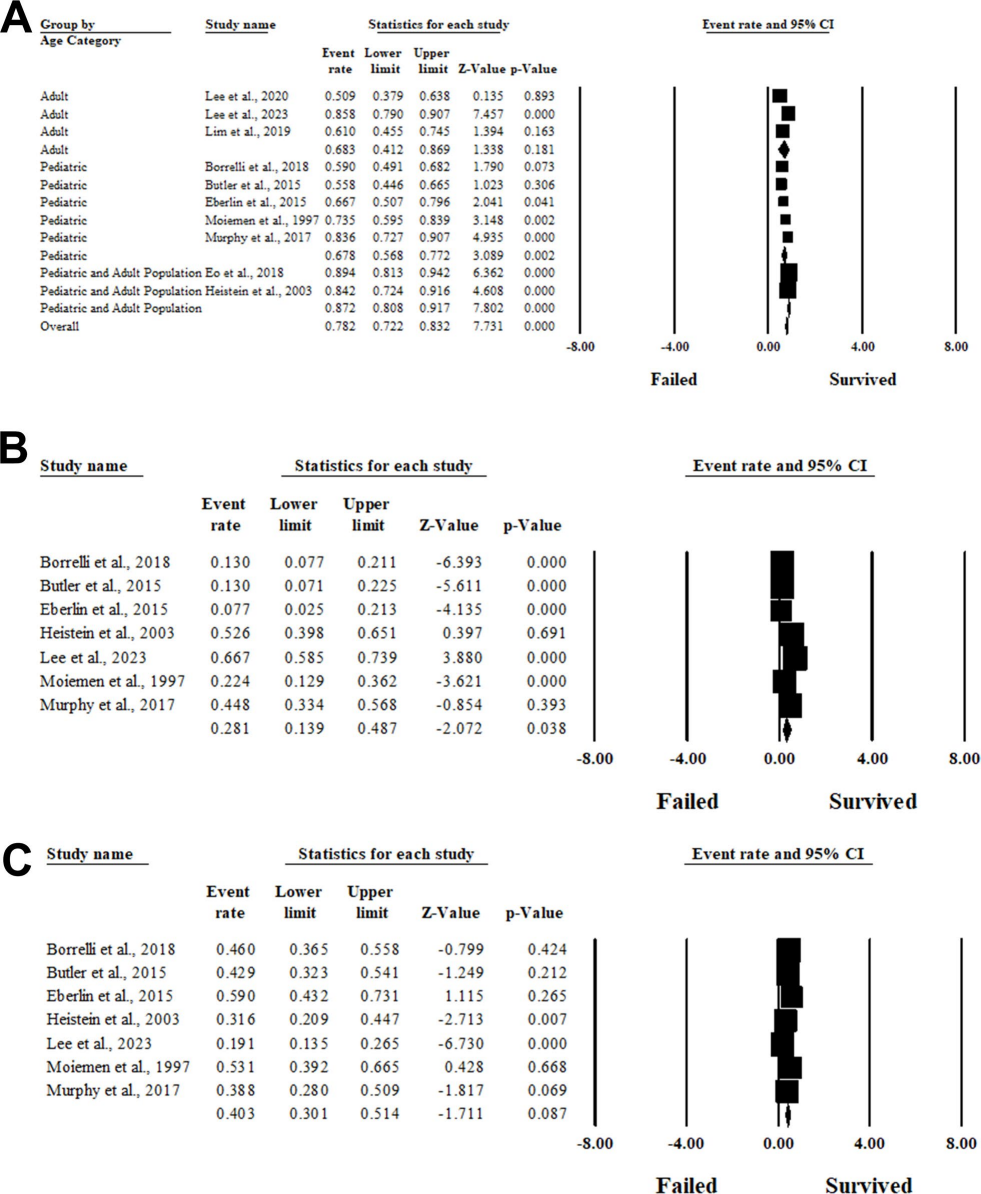
Forest plot of summary analysis of the **A** Event rate and 95% CI of the prevalence of the overall composite graft survivability. **B** The event rate and 95% CI of the prevalence of the complete composite graft survivability. **C** The event rate and 95% CI of the prevalence of the partial composite graft survivability. The size of the black squares is proportional to the statistical weight of each trial. The grey diamond represents the pooled point estimate. The positioning of both diamonds and squares (along with 95% CIs) beyond the vertical line (unit value) suggests a significant outcome

Ten articles included 720 patients with composite graft survival outcomes [7, 21–29]. Out of them, 526 grafts were survived either partially or completely with a pooled overall survivability of 72.8% (95%CI 62.5, 81.1; *p* < 0.001) in the random-effects model (I^2^ = 86.11%, *P* < 0.001). No evidence of publication bias was detected based on the results of Egger’s regression test (Intercept = 6.10, *P* = 0.17). Subgroup analysis based on the pattern of composite graft survivability revealed a complete survival rate of 28.1% with 95%CI ranging from 13.9 to 48.7%, while the partial survival rate was 40.3% (95%CI 30.1, 51.4; *p* < 0.001). (Fig.  2 A, B and C).

## Factors predicting composite graft survivability

### Patients related factors

#### Age

**Fig. 3:**
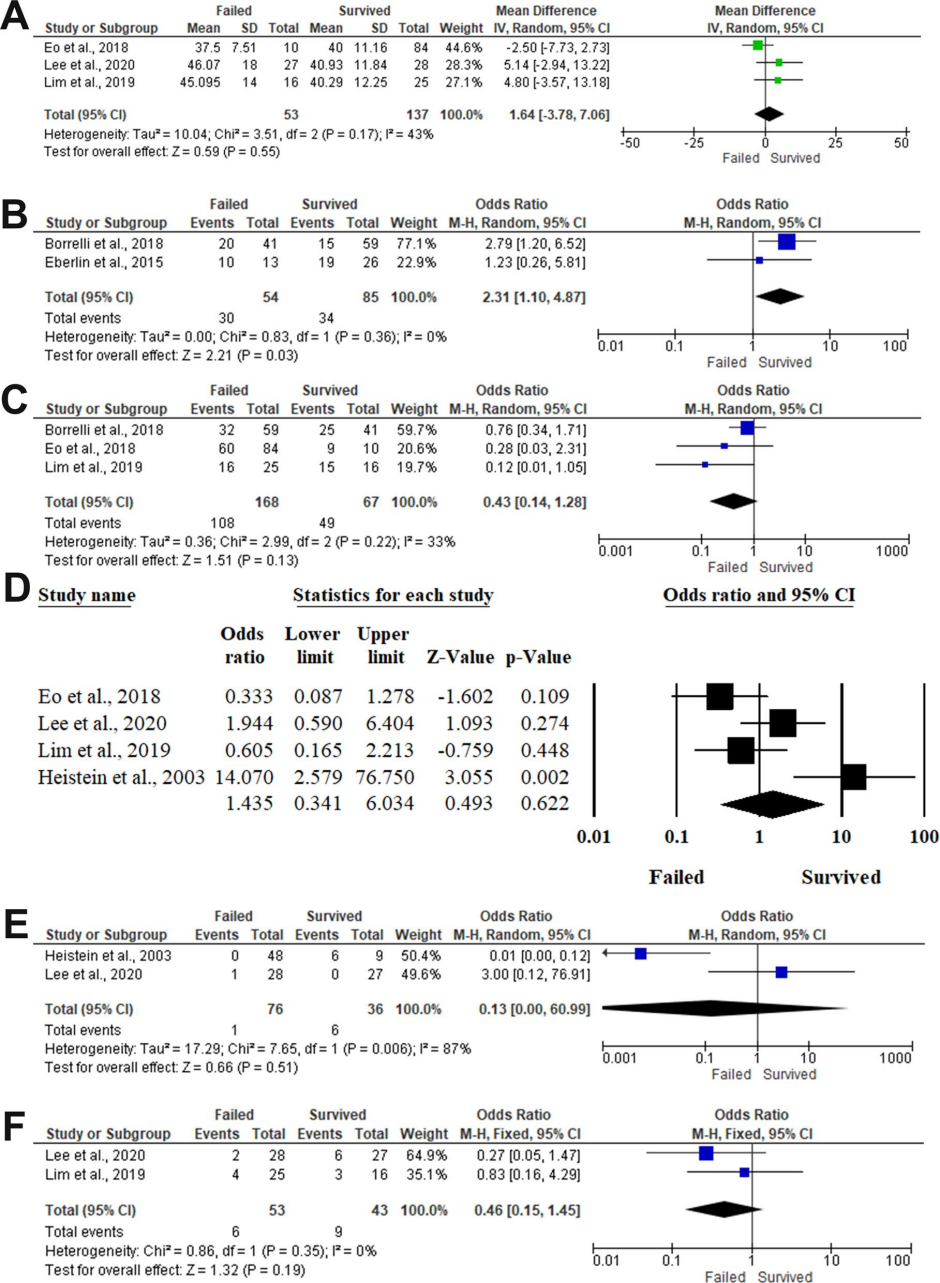
Forest plot of summary analysis of the **A** Mean difference (MD) and 95% CI of the difference in the mean age between survived and failed composite graft groups **B** Odds ratio (OR) and 95% CI of the impact of young age < 4 years on the likelihood of composite graft survival between survived and failed composite graft groups **C** Odds ratio (OR) and 95% CI of the impact of male gender on the likelihood of composite graft survival between survived and failed composite graft groups **D** Odds ratio (OR) and 95% CI of the impact of smoking on the likelihood of composite graft survival between survived and failed composite graft groups **E** Odds ratio (OR) and 95% CI of the impact of diabetes mellitus on the likelihood of composite graft survival between survived and failed composite graft groups. **F** Odds ratio (OR) and 95% CI of the impact of hypertension mellitus on the likelihood of composite graft survival between survived and failed composite graft groups. Size of the green or blue squares is proportional to the statistical weight of each trial. The grey diamond represents the pooled point estimate. The positioning of both diamonds and squares (along with 95% CIs) beyond the vertical line (unit value) suggests a significant outcome (IV = inverse variance)

Three articles included 190 patients with composite grafting, revealed the difference in the mean age between survived and failed composite graft groups [23, 26, 27]. Pooling the data revealed no significant difference between both groups (MD 1.64,95%CI −3.78, 7.06, *P* = 0.55) in the random-effects model (I^2^ = 43%, *P* = 0.17). Consequently, there was a significant association between younger age and composite graft survivability. Particularly, patients aged < 4 years were 2.31 times more susceptible to composite graft take (OR 2.31,95%CI 1.10, 4.87, *P* = 0.03) in the random-effects model (I^2^ = 0%, *P* = 0.36). (Fig. 3 A and B).

#### Gender (Males)

The impact of the male gender on composite graft survivability was assessed in three articles, including 235 patients [21, 23, 27]. There was no statistical association (*P* = 0.13) between male gender and composite graft taken (OR 0.43,95%CI 0.14 to 1.28) with a statistical homogeneity between the included studies (I^2^ = 33%, *P* = 0.22). (Fig. 3C).

#### Comorbidities

##### Smoking

The impact of smoking on the outcomes of composite graft survivability was evaluated in four articles, including 247 patients [23, 24, 26, 27]. In the random-effects model (I^2^ = 72.28%, *P* = 0.013), there was no statistically significant association between smoking and composite graft survivability (OR 1.435,95%CI 0.341 to 6.034, *P* = 0.622). (Fig. 3D).

##### Diabetes mellitus

Two studies, including 112 patients, evaluated the impact of diabetes mellitus on composite graft survivability [24, 26]. There was no statistically significant association (*P* = 0.51) between diabetes mellitus and the composite graft outcomes, with an OR of 0.13, ranging from 0 to 60.99 with a significant heterogeneity between the included studies (I^2^ = 87%, *P* = 0.006). (Fig. 3E).

##### Hypertension

The association between hypertension and composite graft taken was assessed among 96 patients within two articles [26, 27]. There was no statistically significant difference between failed and survived composite grafts groups (OR 0.46,95%CI 0.15, 1.45, *P* = 0.19) with homogeneity between the included studies (I^2^ = 0%, *P* = 0.35). (Fig. 3F).

### Amputation-related factors

#### Time from injury to composite grafting

**Fig. 4:**
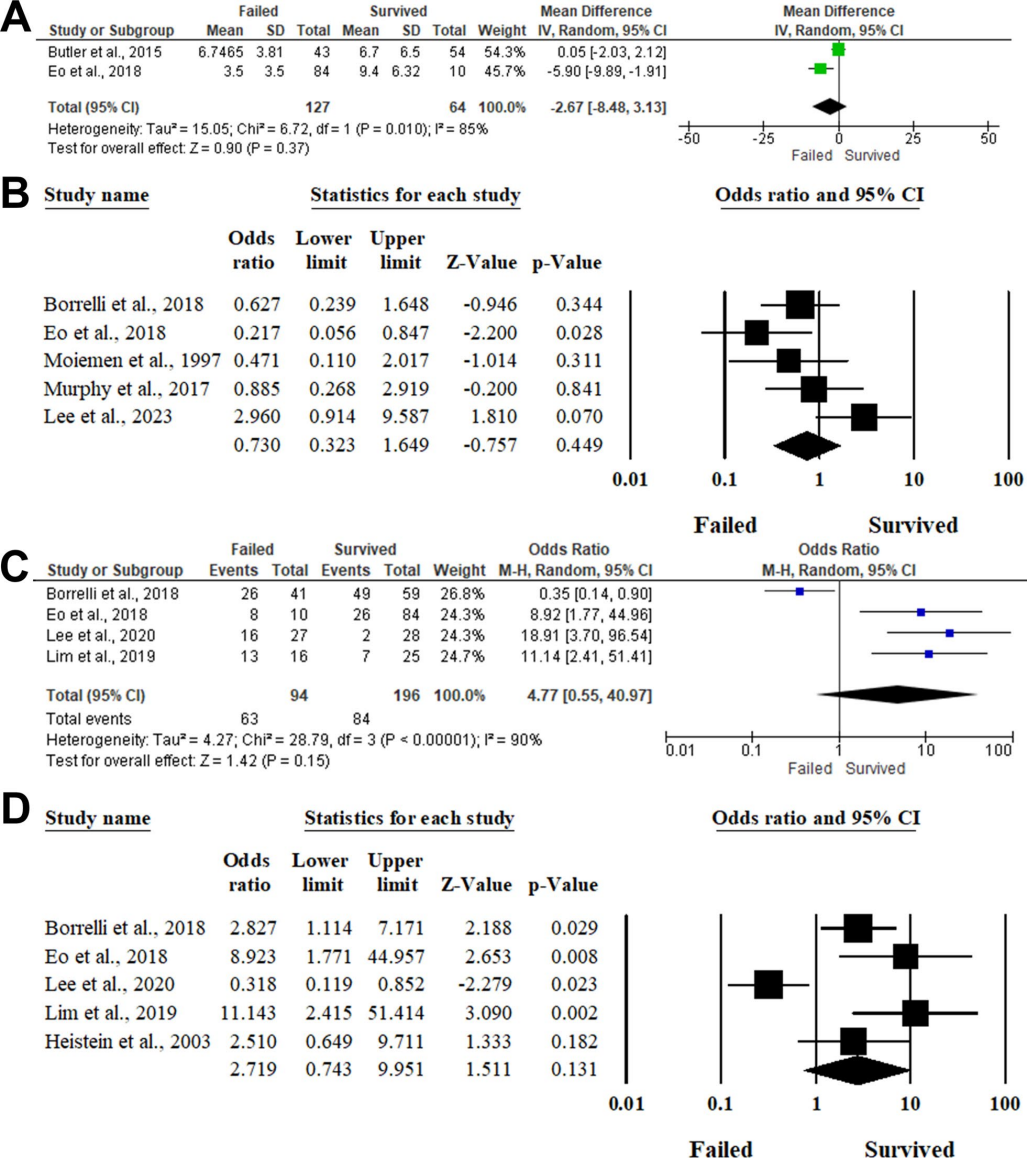
Forest plot of summary analysis of the **A** Mean difference (MD) and 95% CI of the difference in the time from injury to composite grafting between survived and failed composite graft groups **B** Odds ratio (OR) and 95% CI of the impact of time delay > 6 h to composite grafting on the likelihood of composite graft survival between survived and failed composite graft groups **C** Odds ratio (OR) and 95% CI of the impact of crush injuries on the likelihood of composite graft survival between survived and failed composite graft groups **D** Odds ratio (OR) and 95% CI of the impact of cut injuries on the likelihood of composite graft survival between survived and failed composite graft groups**.** Size of the green or blue squares is proportional to the statistical weight of each trial. The grey diamond represents the pooled point estimate. The positioning of both diamonds and squares (along with 95% CIs) beyond the vertical line (unit value) suggests a significant outcome (IV = inverse variance)

Two studies reported the difference between the survived and failed composite grafts regarding the time from injury to composite grafting [7, 23]. There was no statistically significant difference between both groups (MD −2.67,95%CI −8.48, 3.13, *P* = 0.37) with heterogeneity between the included studies (I^2^ = 85%, *P* = 0.010). The association between time delay to composite grafting was evaluated within five studies, including 265 patients [21, 23, 25, 28, 29]. There was no statistically significant association (*P* = 0.449) between composite graft survivability and time delay > 6 h with an OR of 0.730, ranging from 0.323 to 1.649, in the random-effects model (I^2^ = 55.6%, *P* = 0.060). (Fig. 4 A and B).

#### Mechanism of injury

##### Crushing injury

Four articles evaluated the impact of crushing injury on composite graft survivability [21, 23, 26, 27]. There was no statistically significant association between crushing injury and composite graft survivability with an OR of 4.77 and 95%CI; 0.55 to 40.97 with a *P*-Value of 0.15 (I^2^ = 90%, *P* < 0.001). (Fig. 4C**).**

##### Cut injury

Five studies, including 347 patients, assessed the impact of clean-cut amputations on the survival outcomes of composite grafts [21, 23, 24, 26, 27]. Pooling the data in the random-effects model (I^2^ = 81.94%, *P* < 0.001) revealed no significant impact of the cut amputations on the likelihood of composite graft survivability (OR 2.719,95%CI 0.743 to 9.951, *P* = 0.131). (Fig. 4D).

### Levels of amputations

#### Modified-ishikawa classification

##### Level of amputation (1a)

**Fig. 5:**
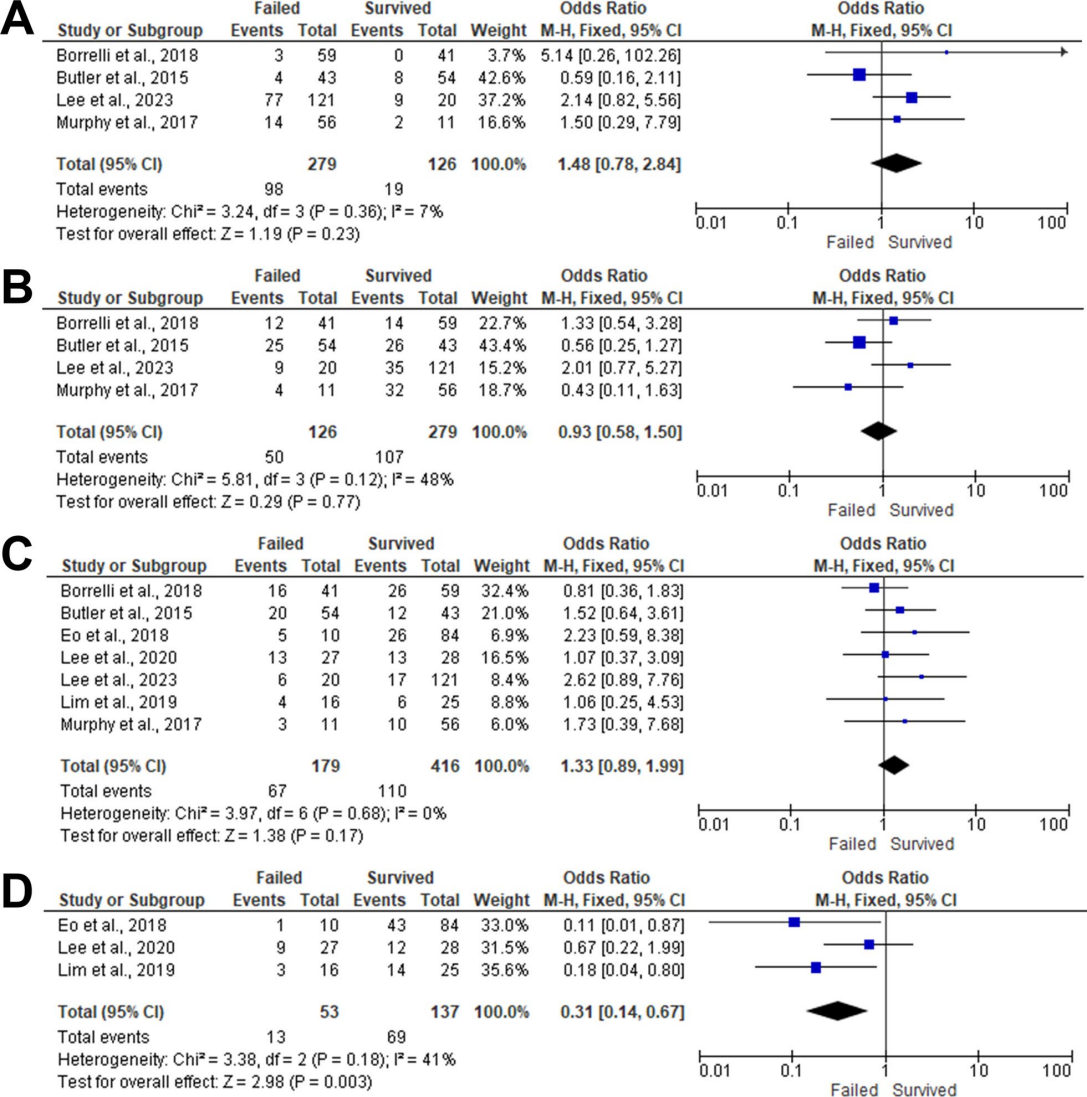
Forest plot of summary analysis of the **A** Odds ratio (OR) and 95% CI of the impact of level of amputation (1a) on the likelihood of composite graft survival between survived and failed composite graft groups **B** Odds ratio (OR) and 95% CI of the impact of level of amputation (1b) on the likelihood of composite graft survival between survived and failed composite graft groups **C** Odds ratio (OR) and 95% CI of the impact of the impact of l the level of amputation (II) on the likelihood of composite graft survival between survived and failed composite graft groups **D** Odds ratio (OR) and 95% CI of the impact of the level of amputation (I) using Das classification on the likelihood of composite graft survival between survived and failed composite graft groups**.** Size of the green or blue squares is proportional to the statistical weight of each trial. The grey diamond represents the pooled point estimate. The positioning of both diamonds and squares (along with 95% CIs) beyond the vertical line (unit value) suggests a significant outcome (IV = inverse variance)

Four articles included 405 patients evaluated the impact of the level of amputation (1a) on the composite graft survivability [7, 21, 25, 29]. There was no statistically significant association between patients with fingertip amputations level 1a and composite graft survivability (OR 1.48,95%CI 0.78 to 2.84, *P* = 0.23) with homogeneity between the included articles (I^2^ = 7%, *P* = 0.36). (Fig. 5A).

##### Level of amputation (1b)

The association between the level of amputation (1b) and composite graft survivability was reported in four articles that included 405 patients [7, 21, 25, 29]. There was no statistically significant association between the level of amputation (1b) and composite graft survivability (OR 0.93,95%CI 0.58, 1.50, *P* = 0.77) with homogeneity between the included articles (I^2^ = 48%, *P* = 0.12). (Fig. 5B).

##### Level of amputation (II)

Seven studies included 177 patients evaluated the impact of the level of amputation (II) on composite graft survivability [7, 21, 23, 25–27, 29]. Pooling the data in the fixed-effect model (I^2^ = 0%, *P* = 0.68) revealed no statistically significant association between level of amputation (II) and composite graft survivability (OR 1.33,95%CI 0.89, 1.99, *P* = 0.17). (Fig. 5C).

### Das classification

#### Level of amputation (I)

Three articles evaluated the impact of the level of amputation (I) using Das classification on the composite graft survivability [23, 26, 27]. Patients with the level of amputation (I) were more susceptible to having a successful composite grafting (OR 0.31,95%CI 0.14 to 0.67, *P* = 0.003) in the fixed-effect model (I^2^ = 47%, *P* = 0.08). (Fig. 5D).

## Discussion

Composite grafting for fingertip amputations is a worthwhile technique in cases where replantation is impossible. The usability of composite grafting has been reported in previous reviews; however, the survival rates and factors associated with composite graft survivability have yet to be investigated conclusively in the literature [9]. The present meta-analysis revealed an overall composite graft survivability of 72.8%, complete survivability of 28.1%, and partial survivability of 40.3%. Composite graft survivability was more pronounced among the pediatric population, particularly patients aged less than four years and patients with amputation level (I). Smokers and patients with crushing injuries were at higher risk of composite graft failure despite not being statistically significant.

Composite graft survivability was associated significantly with the pediatric population, crush injuries, and the level of amputations. These findings were concomitant with Landin et al., 2021 who reported better composite graft outcomes among younger age, more distal amputations, and clean-cut injuries with a graft survivability hovered between 7.7 and 93.5% [9]. Jester et al., 2023 reported a relatively higher composite graft survival rate among the pediatric population, accounting for 81.6%. They attributed composite graft survivability to more distal amputations and shorter time to operation [8]. The central digital artery divides near the end of the fingertip, with more vascularity in this area, supplied by the terminal segmental and fibrous hiatus branches. Furthermore, the reduced volume of detached tissues with more distal amputations required low perfusion with plasma imbibition and capillary inosculation. This could explain the high possibility of composite grafts taken with more distal digital tip amputations [30]. In this respect, Modified Ishikawa classification levels I and II showed no significant association with composite graft survivability, whereas Das classification level I achieved a significant association. Das classification level I included only distal pulp loss with no bony or nail involvement. Paradoxically, Modified Ishikawa classification levels I and II included distal pulp loss in addition to nail and bony involvement with more involvement in level II amputations [31, 32]. Composite grafts incorporate only soft tissue require less metabolic demands to survive, while grafts that incorporate soft tissue, bones and nails require more metabolic demands with less potential to survive[30].

Crush injuries are associated with poor composite graft survivability. This is because of the extensive damage to the vessels, nerves, and soft tissues of the amputated part, as well as the remaining stump. Composite grafts are revascularized via anastomosis between the vessels of the subcutaneous vascular network at the contact area between the graft and the remaining digital stump. This usually occurs within two to five days after grafting. Furthermore, capillary neovascularization occurs after five days of grafting [33, 34]. The non-vascularized composite graft is initially nourished via imbibition, which is affected remarkably with avulsion and crushing injuries. This finding was parallel with Sears et al., 2011 who reported poor survivability among patients with fingertip avulsion injuries who underwent finger replantation [35]. Noteworthy, a failed composite grafting may act as a biological dressing, allowing the underlying wound to re-epithelialize and granulate. This may preserve the digit length, affording more sensations relative to immediate finger terminalization [29].

The present meta-analysis revealed no significant impact of time to composite grafting on the overall survivability. Patients treated with composite grafting before or after six hours after the injury have a similar pattern of graft survivability. Parallel with this finding, Yu et al., 2015 reported a lack of significant impact of ischemia time on the survival rate of finger replantation and highlighted the role of cooling preservation on the overall survivability [36]. Paradoxically, Jester et al., 2023 and Landin et al., 2021 systematic reviews showed a better composite graft taken with a shorter time to grafting [8, 9]. Previous studies identified the time for successful grafting at six hours based on the time at which de-vascularized muscles underwent irreversible ischemia damage. However, fingertip amputations primarily comprise soft tissues that can tolerate up to one day of cold ischemia with fingertip replantation. Appropriate cooling of the amputated fingertip could enhance composite graft survivability. Cooling minimizes the tissue’s metabolic demands and has a bacteriostatic effect, with no further tissue damage. The time delay may have little impact on the composite graft survivability, assuming the graft underwent appropriate cooling preoperatively. Meanwhile, further prospective studies are needed to investigate the impact of time delay intervals on the composite graft survivability in the context of other confounders using the appropriate regression analysis [37].

The present meta-analysis revealed factors influencing the survivability of composite grafting in fingertip amputations. The study included the largest cohort in the literature, assessing predictors of composite graft survivability in different settings and age categories. Conversely, some limitations may negatively impact the resulting evidence. Most of the eligible studies were retrospective, conferring a substantial risk of information bias. Subsequently, there was significant statistical and methodological heterogeneity between the analyzed articles. This heterogeneity may be attributed to the significant variation between the analyzed articles regarding the recruitment criteria, sample sizes, follow-up period, definition of the level of the amputation, recognizing graft success, and demographic characteristics of the included patients. The resulting statistical heterogeneity was resolved by applying the random-effects model and doing subgroup analysis. Further prospective studies with adequate samples and prolonged follow-up periods are necessary to mitigate the potential limitations of the analyzed studies.

## Conclusions

Composite grafting is a feasible and effective procedure for restoring aesthetically functional digits among patients with traumatically amputated fingertips. The composite graft survived among the majority of the patients, with a more significant survival pattern among younger populations and patients with more distal amputations. Subsequently, patients with crushing injuries, more proximal amputations, and smokers were at higher risk of composite graft failure. Identifying factors associated with composite graft survivability could help hand surgeons identify vulnerable populations early and implement preventive measures to minimize the risk of graft failure in patients with fingertip amputations.

## Supplementary Information

Supplementary file 1.

## Data Availability

The datasets used in the present study are available from the first author and corresponding authors on reasonable request.

## Abbreviations

PRISMA: preferred reporting items for systematic reviews and meta-analysis
MD: mean difference
SMD: standardized mean difference
CI: confidence interval
