# Supplementary material for: Factors predicting composite grafts survivability in patients with fingertip amputations; a systematic review and meta-analysis
Source: J Orthop Surg Res. 2024 Nov 18;19:765. doi: 10.1186/s13018-024-05230-9 (PMC11572166; doi:10.1186/s13018-024-05230-9)
Supplement: Supplementary file 1 [file 13018_2024_5230_MOESM1_ESM.docx]

| **Table. S1 PRISMA 2009. Checklist** | | | |
| --- | --- | --- | --- |
| **Section/topic** | **#** | **Checklist item** | **Reported on page #** |
| **TITLE** | | |  |
| Title | 1 | Identify the report as a systematic review, meta-analysis, or both. | 1 |
| **ABSTRACT** | | |  |
| Structured summary | 2 | Provide a structured summary including, as applicable: background; objectives; data sources; study eligibility criteria, participants, and interventions; study appraisal and synthesis methods; results; limitations; conclusions and implications of key findings; systematic review registration number. | 2 |
| **INTRODUCTION** | | |  |
| Rationale | 3 | Describe the rationale for the review in the context of what is already known. | 3-4 |
| Objectives | 4 | Provide an explicit statement of questions being addressed with reference to participants, interventions, comparisons, outcomes, and study design (PICOS). | 4 |
| **METHODS** | | |  |
| Protocol and registration | 5 | Indicate if a review protocol exists, if and where it can be accessed (e.g., Web address), and, if available, provide registration information including registration number. | 4 |
| Eligibility criteria | 6 | Specify study characteristics (e.g., PICOS, length of follow-up) and report characteristics (e.g., years considered, language, publication status) used as criteria for eligibility, giving rationale. | 5 |
| Information sources | 7 | Describe all information sources (e.g., databases with dates of coverage, contact with study authors to identify additional studies) in the search and date last searched. | 5 |
| Search | 8 | Present full electronic search strategy for at least one database, including any limits used, such that it could be repeated. | 5 |
| Study selection | 9 | State the process for selecting studies (i.e., screening, eligibility, included in systematic review, and, if applicable, included in the meta-analysis). | 5 |
| Data collection process | 10 | Describe method of data extraction from reports (e.g., piloted forms, independently, in duplicate) and any processes for obtaining and confirming data from investigators. | 6 |
| Data items | 11 | List and define all variables for which data were sought (e.g., PICOS, funding sources) and any assumptions and simplifications made. | 6 |
| Risk of bias in individual studies | 12 | Describe methods used for assessing risk of bias of individual studies (including specification of whether this was done at the study or outcome level), and how this information is to be used in any data synthesis. | 6 |
| Summary measures | 13 | State the principal summary measures (e.g., risk ratio, difference in means). | 6 |
| Synthesis of results | 14 | Describe the methods of handling data and combining results of studies, if done, including measures of consistency (e.g., I^2^) for each meta-analysis. | 6-7 |

Page 1 of 2

| **Section/topic** | **#** | **Checklist item** | **Reported on page #** |
| --- | --- | --- | --- |
| Risk of bias across studies | 15 | Specify any assessment of risk of bias that may affect the cumulative evidence (e.g., publication bias, selective reporting within studies). | 6 |
| Additional analyses | 16 | Describe methods of additional analyses (e.g., sensitivity or subgroup analyses, meta-regression), if done, indicating which were pre-specified. | 7 |
| **RESULTS** | | |  |
| Study selection | 17 | Give numbers of studies screened, assessed for eligibility, and included in the review, with reasons for exclusions at each stage, ideally with a flow diagram. | 7 |
| Study characteristics | 18 | For each study, present characteristics for which data were extracted (e.g., study size, PICOS, follow-up period) and provide the citations. | 7-8 |
| Risk of bias within studies | 19 | Present data on risk of bias of each study and, if available, any outcome level assessment (see item 12). | 8 and table.2 |
| Results of individual studies | 20 | For all outcomes considered (benefits or harms), present, for each study: (a) simple summary data for each intervention group (b) effect estimates and confidence intervals, ideally with a forest plot. | 8-13 and tables 1 and 2 |
| Synthesis of results | 21 | Present results of each meta-analysis done, including confidence intervals and measures of consistency. | 8-13 and Figures 2-5 |
| Risk of bias across studies | 22 | Present results of any assessment of risk of bias across studies (see Item 15). | Table.2 |
| Additional analysis | 23 | Give results of additional analyses, if done (e.g., sensitivity or subgroup analyses, meta-regression [see Item 16]). | 8 and Figure.2 |
| **DISCUSSION** | | |  |
| Summary of evidence | 24 | Summarize the main findings including the strength of evidence for each main outcome; consider their relevance to key groups (e.g., healthcare providers, users, and policy makers). | 13 |
| Limitations | 25 | Discuss limitations at study and outcome level (e.g., risk of bias), and at review-level (e.g., incomplete retrieval of identified research, reporting bias). | 15 |
| Conclusions | 26 | Provide a general interpretation of the results in the context of other evidence, and implications for future research. | 16 |
| **FUNDING** | | |  |
| Funding | 27 | Describe sources of funding for the systematic review and other support (e.g., supply of data); role of funders for the systematic review. | Title Page |

| **Supplementary Table 2. The search strategy for the included databases** | | | | | |
| --- | --- | --- | --- | --- | --- |
|  | **Database** | **Date** | **Keywords** | **Results** | **Link** |
| **1** | **PubMed** | 24-7-2023 | ((((((((((fingertip[Title]) OR (fingertips[Title])) OR (digital tip[Title])) OR (digital tips[Title])) OR (digit[Title])) OR (digits[Title])) OR (finger[Title])) OR (fingers[Title])) OR (thumb[Title])) OR (thumbs[Title])) AND (Graft[Title]) | 189 | [Link](https://pubmed.ncbi.nlm.nih.gov/?term=%28%28%28%28%28%28%28%28%28%28fingertip%5BTitle%5D%29+OR+%28fingertips%5BTitle%5D%29%29+OR+%28digital+tip%5BTitle%5D%29%29+OR+%28digital+tips%5BTitle%5D%29%29+OR+%28digit%5BTitle%5D%29%29+OR+%28digits%5BTitle%5D%29%29+OR+%28finger%5BTitle%5D%29%29+OR+%28fingers%5BTitle%5D%29%29+OR+%28thumb%5BTitle%5D%29%29+OR+%28thumbs%5BTitle%5D%29%29+AND+%28Graft%5BTitle%5D%29&sort=pubdate) |
|  |  |  | ((((((((((fingertip[Title]) OR (fingertips[Title])) OR (digital tip[Title])) OR (digital tips[Title])) OR (digit[Title])) OR (digits[Title])) OR (finger[Title])) OR (fingers[Title])) OR (thumb[Title])) OR (thumbs[Title])) AND (Grafts[Title]) | 99 | [Link](https://pubmed.ncbi.nlm.nih.gov/?term=%28%28%28%28%28%28%28%28%28%28fingertip%5BTitle%5D%29+OR+%28fingertips%5BTitle%5D%29%29+OR+%28digital+tip%5BTitle%5D%29%29+OR+%28digital+tips%5BTitle%5D%29%29+OR+%28digit%5BTitle%5D%29%29+OR+%28digits%5BTitle%5D%29%29+OR+%28finger%5BTitle%5D%29%29+OR+%28fingers%5BTitle%5D%29%29+OR+%28thumb%5BTitle%5D%29%29+OR+%28thumbs%5BTitle%5D%29%29+AND+%28Grafts%5BTitle%5D%29&sort=pubdate) |
| **2** | **Google Scholar** | 24-7-2023 | allintitle: Graft fingertip OR fingertips OR "digital tip" OR "digital tips" OR digit OR digits OR finger OR fingers OR thumb OR thumbs | 351 | [Link](https://scholar.google.com/scholar?as_q=Graft&as_epq=&as_oq=fingertip+fingertips+%22digital+tip%22+%22digital+tips%22+digit+digits+finger+fingers+thumb+thumbs&as_eq=&as_occt=title&as_sauthors=&as_publication=&as_ylo=&as_yhi=&hl=en&as_sdt=0%2C5) |
|  |  |  | allintitle: Grafts fingertip OR fingertips OR "digital tip" OR "digital tips" OR digit OR digits OR finger OR fingers OR thumb OR thumbs | 171 | [Link](https://scholar.google.com/scholar?hl=en&as_sdt=0%2C5&q=allintitle%3A+Grafts+fingertip+OR+fingertips+OR+%22digital+tip%22+OR+%22digital+tips%22+OR+digit+OR+digits+OR+finger+OR+fingers+OR+thumb+OR+thumbs&btnG=) |
| **3** | **Web of Science** | 24-7-2023 | "fingertip " OR "fingertips " OR " digital tip" OR " digital tips" OR " digit" OR "digits" OR " finger" OR "fingers" OR "thumb" OR "thumbs" (Title) AND "Graft" OR "Grafts" (Title) | 286 | [Link](https://0810o261d-1106-y-https-www-webofscience-com.mplbci.ekb.eg/wos/woscc/summary/017d8e6f-aff3-4e99-ba90-749edd2b3e37-99b469d2/relevance/1) |
| **4** | **Scopus** | 24-7-2023 | - ( TITLE ( fingertip ) OR TITLE ( fingertips ) OR TITLE ( digital AND tip ) OR TITLE ( digital AND tips ) OR TITLE ( digit ) OR TITLE ( digits ) OR TITLE ( finger ) OR TITLE ( fingers ) OR TITLE ( thumb ) OR TITLE ( thumbs ) AND TITLE ( graft ) OR TITLE ( grafts ) ) | 311 | [Link](https://08105263b-1106-y-https-www-scopus-com.mplbci.ekb.eg/results/results.uri?sort=plf-f&src=s&st1=fingertip&st2=fingertips&searchTerms=digital+tip%3F%21%22*%24digital+tips%3F%21%22*%24digit%3F%21%22*%24digits%3F%21%22*%24finger%3F%21%22*%24fingers%3F%21%22*%24thumb%3F%21%22*%24thumbs%3F%21%22*%24Graft%3F%21%22*%24Grafts%3F%21%22*%24&sid=84920c7058a0668b3e1b2ca7dc551131&sot=b&sdt=b&sl=219&s=%28TITLE%28fingertip%29+OR+TITLE%28fingertips%29+OR+TITLE%28digital+tip%29+OR+TITLE%28digital+tips%29+OR+TITLE%28digit%29+OR+TITLE%28digits%29+OR+TITLE%28finger%29+OR+TITLE%28fingers%29+OR+TITLE%28thumb%29+OR+TITLE%28thumbs%29+AND+TITLE%28Graft%29+OR+TITLE%28Grafts%29%29&origin=searchbasic&editSaveSearch=&yearFrom=Before+1960&yearTo=Present&sessionSearchId=84920c7058a0668b3e1b2ca7dc551131&limit=10) |
| **5** | **EMBASE** | 24-7-2023 | (fingertip:ti OR fingertips:ti OR 'digital tip':ti OR 'digital tips':ti OR digit:ti OR digits:ti OR finger:ti OR fingers:ti OR thumb:ti OR thumbs:ti) AND graft:ti | 217 | [Link](https://www.embase.com/#advancedSearch/resultspage/history.1/page.1/200.items/orderby.date/source.) |
|  |  |  | (fingertip:ti OR fingertips:ti OR 'digital tip':ti OR 'digital tips':ti OR digit:ti OR digits:ti OR finger:ti OR fingers:ti OR thumb:ti OR thumbs:ti) AND grafts:ti | 119 | [Link](https://www.embase.com/#advancedSearch/resultspage/history.2/page.1/200.items/orderby.date/source.) |
| **6** | **SIGLE** | 24-7-2023 | "fingertip " OR "fingertips " OR " digital tip" OR " digital tips" OR " digit" OR "digits" OR " finger" OR "fingers" OR "thumb" OR "thumbs" AND "Graft" OR "Grafts" | 0 | [Link](https://opengrey.eu/?s=%22fingertip+%22+OR+%22fingertips+%22+OR+%22+digital+tip%22+OR+%22+digital+tips%22+OR+%22+digit%22+OR+%22digits%22+OR+%22+finger%22+OR+%22fingers%22+OR+%22thumb%22+OR+%22thumbs%22+++AND+%22Graft%22+OR+%22Grafts%22) |
| **7** | **Virtual Health Library** | 24-7-2023 | (ti:(fingertip)) OR (ti:(fingertips)) OR (ti:(digital tip)) OR (ti:(digital tips)) OR (ti:(digit)) OR (ti:(digits)) OR (ti:(Finger)) OR (ti:(Fingers)) OR (ti:(Thumb)) OR (ti:(Thumbs)) AND (ti:(Graft)) | 1 | [Link](https://pesquisa.bvsalud.org/portal/?output=site&lang=en&from=0&sort=&format=summary&count=20&fb=&page=1&skfp=&index=&q=%28ti%3A%28fingertip%29%29+OR+%28ti%3A%28fingertips%29%29+OR+%28ti%3A%28digital+tip%29%29+OR+%28ti%3A%28digital+tips%29%29+OR+%28ti%3A%28digit%29%29+OR+%28ti%3A%28digits%29%29+OR+%28ti%3A%28Finger%29%29+OR+%28ti%3A%28Fingers%29%29+OR+%28ti%3A%28Thumb%29%29+OR+%28ti%3A%28Thumbs%29%29+AND+%28ti%3A%28Graft%29%29+&search_form_submit=) |
| **8** | **NYAM** | 24-7-2023 | "fingertip " OR "fingertips " OR " digital tip" OR " digital tips" OR " digit" OR "digits" OR " finger" OR "fingers" OR "thumb" OR "thumbs" AND "Graft" OR "Grafts" | 0 | [Link](http://www.greylit.org/library/search?fbclid=IwAR0jWQma941_Bqn0v10yaWh2vIGdN_-Jm2Sl0wz22EfkHZf6x1nFgyTppFU#wt=json&facet=true&q=Botulinum%20%20AND%20%20Oncoplastic%20OR%20Mammoplasty%20OR%20Breast%20OR%20%22Breast%20Reduction%22%20OR%20%22%20Breast%20Augmentation%22%20OR%20Breast%20OR%20Pectoral%20OR%20Pectoralis%20OR%20Mammaplasty&q.op=AND&fl=id&qt=dismax&sort=created%20desc&page=1&per_page=10&start=0&qf=full_text&facet.field=publisher&facet.field=full_subjects) |
| **9** | **Clinical Trials.Gov** | 24-7-2023 | **Finger \| Graft** | 6 | [Link](https://classic.clinicaltrials.gov/ct2/results?cond=Finger&term=&type=&rslt=&age_v=&gndr=&intr=Graft&titles=&outc=&spons=&lead=&id=&cntry=&state=&city=&dist=&locn=&rsub=&strd_s=&strd_e=&prcd_s=&prcd_e=&sfpd_s=&sfpd_e=&rfpd_s=&rfpd_e=&lupd_s=&lupd_e=&sort=) |
| **10** | **Controlled Trials (mRCT)** | 24-7-2023 | "( Interventions: Graft AND Condition: Finger )" | 0 | [Link](https://www.isrctn.com/search?q=&filters=condition%3AFinger%2Cintervention%3AGraft) |
| **11** | **ICTRP** | 24-7-2023 | Finger AND Graft | 1 | [Link](https://trialsearch.who.int/AdvSearch.aspx) |
| **12** | **Cochrane Library** | 24-7-2023 | Finger in Record Title AND Graft in Record Title - (Word variations have been searched) | 4 | [Link](https://0812826ix-1106-y-https-www-cochranelibrary-com.mplbci.ekb.eg/advanced-search?cookiesEnabled) |

| **Supplementary Table.3** Classifications of fingertip amputations | |
| --- | --- |
| **Modified-Ishikawa Classification [1]** | |
| I | Distal to midnail |
| **Ia** | beyond the distal edge of the nail |
| **Ib** | between the midnail and distal edge of the nail) |
| II | Between midnail and nail base (eponychium) |
| III | Midway between eponychium and distal interphalangeal joint (DIPJ) |
| IV | Between II and DIPJ |
| **Das Classification[2]** | |
| Type I | Only distal pulp loss |
| Type II | Loss of pulp and terminal phalanx up to the distal one-third |
| Type III | Loss of pulp and terminal phalanx up to the distal three-fourths |
| **Allen’s classification[3]** | |
| Type I | Loss of only the pulp of the finger |
| Type II | Pulp and nail loss without bone fragment in the distal amputated fingertip |
| Type III | Partial loss of the distal phalanx plus corresponding loss of the pulp and nail |
| Type IV | Loss proximal to the germinal matrix |

**Refrences**

1. Venkatesh, A., A. Khajuria, and A. Greig, *Management of pediatric distal fingertip injuries: a systematic literature review.* Plastic and Reconstructive Surgery–Global Open, 2020. **8**(1): p. e2595.

2. Das, S.K. and H. Brown, *Management of lost finger tips in children.* Hand, 1978(1): p. 16-27.

3. Peterson, S.L., E.L. Peterson, and M.J. Wheatley, *Management of fingertip amputations.* The Journal of hand surgery, 2014. **39**(10): p. 2093-2101.
